# Application of impedance measurement to investigate in vitro inhalation toxicity of bacteria

**DOI:** 10.1186/s12995-021-00317-z

**Published:** 2021-08-12

**Authors:** Stefanie Klar, Dierk-Christoph Poether, Jessica Reinert, Nicole Hüttig, Gunter Linsel, Udo Jäckel

**Affiliations:** Federal Institute for Occupational Safety and Health, Nöldnerstraße 40-42, 10317 Berlin, Germany

**Keywords:** ECIS, Cytotoxicity, Bacteria, Occupational health, In vitro, NuLi-1, Lung epithelial cells

## Abstract

**Background:**

Workers of agriculture and intensive life stock farming are exposed to highly contaminated workplaces. Bioaerosol exposures are suspected to trigger respiratory health effects of the workers. So far, risk evaluation of bioaerosols has been assessed through the infectivity of comprising biological agents that is classified in Europe by four risk groups according to the criteria of Directive 2000/54EC of the European Parliament. However, this directive additionally requires the risk assessment of allergenic and toxigenic effects without further elaboration. The aim of our study was to establish an in vitro screening system that is able to measure inhalative toxic effects of bacteria and their metabolites.

**Methods:**

In this study, we analyzed three bacterial toxins and five culture supernatants of selected bacteria with known toxicity as model agents exposed to the lung epithelial cell line NuLi-1. We used electrical cell-substrate impedance sensing (ECIS) method to monitor real-time cell changes and the viability test Prestoblue™.

**Results:**

We confirmed concentration dependent cytotoxic effects of the selected toxins in NuLi-1 cells over a period of up to 48 h. Each toxin resulted in a different but specific impedance profile over time according to their mode of action, whereas viability assay showed the metabolic activity of the cells at a chosen time point without revealing any information on their mode of action. Furthermore, dose-response-relationships were monitored. Tested model bacteria (*Streptoccous pneumoniae, Acinetobacter radioresistens, Aerococcus viridans, Aeromonas hydrophila*) reacted according to their expected toxicity except one bacterium (*Enterococcus faecalis*). The established assays revealed the concentration dependent onset and intensity of bacterial cytotoxicity and the viability of the cells at 24 h and 48 h exposure.

**Conclusion:**

Impedance measurement and the viability assay Prestoblue™ in combination are suitable as sensitive screening methods to analyze toxic potential of bacteria and can therefor support the risk assessment of workplaces in terms of the directive 2000/54/EC.

## Introduction

Employees should be protected from risks caused by biological agents at their workplaces applying the strategy of risk assessment (Directive 2000/54/EC).

The risk assessment needs to comprise the infectious, toxigenic and allergenic potential of biological agents. For the infectious risk assessment, biological agents are separated into risk groups. However, the assessment of the allergenic and toxic potential of biological agent is not sufficiently feasible and not covered by the classification into the established risk groups. In order to protect workers sufficiently, it is essential to assess the sensitizing and toxic potential of the biological agents in addition to their infectious potential. At workplaces with biological security levels, e.g., in laboratories or health facilities, biological agents are known in most cases and protective measures are based on their risk group. However, in case of agriculture, waste management, etc., the assessment is much more difficult, as the composition of biological agents is much more complex and varies widely. Diseases of toxic and allergenic causes play a major role in addition to infectious diseases [1]. Nonetheless, in Germany, there is no commitment to measure biological agents at workplaces.

Up to now, the toxic potential of biological agents is often estimated exclusively by the endotoxin content and has been reviewed in several publications [2-4], although other components influence the toxic potential as well. These include, for example, exotoxins of the bacteria and mycotoxins of molds. They are secreted in the course of secondary metabolism and are not vital for the microorganisms. Exotoxins are usually proteins, while mycotoxins belong to a wide variety of chemical substances. These toxins can act in very different ways.

A known bacterial toxin, which has a health adverse effect in the lungs, is the pertussis toxin from *Bordetella pertussis*. It acts on the epithelial cells of the lungs and impairs their barrier function and furthermore the innate immune response [5]. Another example, the alpha-toxin of *Staphylococcus aureus* is also involved in pneumonia and attacks pulmonary epithelial cells. The toxin forms transmembrane pores, which leads to the collapse of the cells and epithelial cells can no longer exercise their barrier function [6]. These are just two examples of many bacterial toxins that can have an impact on the lungs. Lung epithelial cells represent the first row of host defense and biological barrier against potentially harmful substances as well as microorganisms. The toxic effects range from a regulatory disturbance, e.g., inflammatory effects caused by endotoxins, to necrotic or apoptotic cell destruction [7]. In order to be able to assess the toxicity of biological agents, it is important to establish standardized methods providing information on possible cellular effects. Among many in vitro methods, impedance measurement is a promising label-free, non-destructive real-time method for detecting cell responses to e.g., toxins of chemical or biological origin. This method was primarily used to study the adhesion of cultured cell lines [8]. The “electric cell-substrate impedance sensing” (ECIS) method is based on cells growing adhered to small gold electrodes located at the bottom of cell culture dishes. Cells act as dielectric layers which results in a measurable cell-specific impedance. Cytotoxic substances affect cells and thus changes in the impedance can be monitored. ECIS has been intensively tested and is routinely used to analyze e.g., the adherence and distribution of cells, cell movements, cell-cell contacts, cell proliferation and in vitro toxicology in real time [9].

Some studies have shown that half-maximum effective concentrations obtained with ECIS match the values of other cytotoxicity assays such as neutral red uptake (NRU), (3-(4,5-dimethylthiazol-2-yl)-2,5-diphenyltetrazolium bromide (MTT) test, lactate dehydrogenase (LDH) measurement, [10-12].

It is well known that occupational inhalational exposure to bioaerosols of high microbial load could lead to respiratory disease like inflammation, allergies and infections [13, 14]. For our studies, we choose the NuLi-1 cell line, which is often used for in vitro studies of cystic fibrosis [15]. The cell line display several of the primary culture properties and exhibit characteristics of a polarized differentiated epithelium in air liquid interface (ALI) cultivation [16, 17]. NuLi-1 cells are suitable for studying innate immunity and have also been used to study mechanisms of bacterial infection and inflammation [18-20]. More importantly, this cell line is hTERT-transfected and of human origin, thereby overcoming the constraints of using primary cells, like limited passage number, and donor dependent heterogeneity.

In this study, the ECIS method in combination with a viability assay was applied to investigate the response of the human lung epithelial cell line NuLi-1 to bacterial toxins and culture supernatant. To verify the toxic impact three bacterial toxins (Streptolysin-O, Hygromycin B and Nigericin) have been tested. Streptolysin O is a pore-forming hemolytic exotoxin. Hygromycin B is an antibiotic produced by the bacterium *Streptomyces hygroscopicus* inhibiting protein synthesis and Nigericin also produced by *Streptomyces hygroscopicus* is an ionophore (antiporter of H^+^ and K^+^). Hereinafter, analysis of culture supernatants of four bacteria with known toxicity (*Streptococcus pneumoniae, Acinetobacter radioresistens, Enterococcus faecalis, Aeromonas hydrophila)* were performed. *S. pneumoniae* and *A. hydrophila* are known as toxin-producers whose toxins have a cell-damaging effect by perforating the membrane of host cells [21, 22]. *A. radioresistens* is known for lacing vesicles, so-called “outer membrane vesicles (OMV)”, which may play a role in the induction of apoptosis [23, 24]. *E. faecalis* is a bacterium found in the intestine and highly virulent strains are able to form a cytolysine [25]. In order to investigate a relatively unknown bacterium in this experimental setup, we also studied the properties of *A. viridans*, which was often found in intensive animal husbandry facilities [26].

## Material and methods

### Cell culture

NuLi-1 cells (ATCC-CRL-4011) were obtained from the American Type Culture Collection (ATCC, Virginia, USA). The hTERT (human telomerase reverse transcriptase)-transfected cell line derived from 36-year-old healthy male human donor. Cells do not undergo growth arrest in cell culture due to retroviral expression of the telomerase and human papilloma virus-16 E6/E7 genes. They were grown in supplemented serum-free airway epithelial cell basal medium (AECBM; ATCC-PCS-300-040; ATCC-PCS-300-030). Cells were culture in CELLBIND cell culture flasks (Corning®, NY, USA) under standard conditions (37 °C; 5% CO_2_). Only cells between passages 4 and 11 were used for experiments.

### Toxins

Toxins were obtained from the following manufacturers: Streptolysin O from Sigma-Aldrich (S5265; Saint Louis, Missouri, USA); Nigericin from Invivogen (tlrl-nig; San Diego, USA) and Hygromycin B from Roche (H777–2; Mannheim, Germany). All toxins were dissolved and stored according to manufacturer’s specifications.

### Bacteria

*Streptococcus pneumoniae* (NCTC7466)*, Acinetobacter radioresistens* (DSMZ 6976)*, Enterococcus faecalis* (DSMZ 20478)*, Aeromonas hydrophila* (DSMZ 30187) and *Aerococcus viridans* (DSMZ 20340) were acquired from the National Collection of Type Cultures (NCTC, Salisbury, UK) and the German Collection of Microorganisms and Cell Culture GmbH (DSMZ; Braunschweig, Germany). All bacteria were cultured in Todd Hewitt Broth + 0.5% yeast extract (THY) medium overnight until stationary growth phase. Subsequently, the colony-forming units were determined per ml (cfu/ml) summarized in Table 1. For toxicity testing, cultures were centrifuged (15.000 x g) to separate bacteria from culture supernatants. Culture supernatants were filtered (0.2 μm sterile filter) and stored until use at − 80 °C. For experiments, four different dilutions of the sterile culture supernatants were used.

**Table 1:** Corresponding concentrations (colony forming units (cfu)/ml) of the used bacterial culture supernatants

Bacteria	cfu/ml
*E. faecalis*	2.40E+08
*A. radioresistens*	1.80E+09
*S. pneumoniae*	3.40E+07
*A. viridans*	1.50E+08
*A. hydrophila*	3.70E+09

### Impedance measurements and data processing

Impedance measurements were carried out using the ECIS system (ECIS-Z Theta; Applied Biophysics; New York, USA) equipped with a 96-well station. In order to produce a confluent monolayer of NuLi-1 cells for the ECIS assay, cells were seeded at a concentration of 1 × 10^4^ cells /well in a 96 well cell culture test plate (96idf10e, Applied Biophysics). For this purpose, the wells were previously coated with 100 μl collagen IV solution (Sigma Aldrich; c = 60 μg/ml) for 60 min at room temperature. Afterwards, collagen was removed and replaced by 100 μl AECBM medium. Subsequently, 200 μl cell suspension per well was added and cells were incubated for 48 h at 37 °C/ 5% CO_2_ until a confluent monolayer was reached which was confirmed by a plateau of the impedance measurement. For exposure, 200 μl of medium was removed. In addition to the remaining 100 μl medium, 100 μl fresh medium and 100 μl sample were added (total volume per well 300 μl). ECIS assay was carried out at frequencies of 4, 16 and 64 kHz for each well over a measuring period for another 48 h. Measurements were set up in the minimum time interval allowed by the 96-well plate which means every single well was measured repeatedly every 4 min. The data were analyzed using the Applied Biophysics Z-theta Analysis Software v 1.2.92.0. To analyze ECIS results, impedance values were normalized using the analysis software by subtracting each measured value (n) by the initial value (n_0_), n-n_0_ thus all values become zero at the time point when samples were added. Sample exposed cells where then compared to media only control cells. The Graph Pad Prism Software was used to graphically represent the impedance values reflecting cell monolayer integrity and overall response to the samples over the period of measurement of 48 h at 6 h intervals and differences between the supernatant exposed data and control data are shown.

### Viability assay

Viability of NuLi-1 cells was determined by the resazurin-based PrestoBlue™ assay (Invitrogen; A13262; Carlsbad, USA). Non-fluorescent resazurin is reduced to highly fluorescent resofurin in metabolic active cells. For the experiments, 1 × 10^4^ cells/well on a 96-well microtiter plate (Cellbind clear flat bottom, black, Corning) were seeded and incubated for 48 h at 37 °C/ 5% CO_2_ until a confluent monolayer was reached. At the beginning of the exposure, 200 μl of medium were removed. In addition to the remaining 100 μl medium, 100 μl of fresh medium and 100 μl sample were added (total volume per well 300 μl) in triplicates incubated for further 24 and 48 h respectively. After exposure time, supernatants were removed and 10% Prestoblue™ solution in AECBM was added to each approach and incubated for additional 2 h. Changes in cell viability was measured by fluorescence spectroscopy (excitation:570 nm/ emission:610 nm). Viability was expressed as a percentage relative to the negative control.

### Statistical analysis

Mean values and standard deviation of three independent experiments are reported. Statistical analysis was performed using t-tests in Graph Pad Prism (Version 8). Values of *p* < 0.05 were considered statistically significant. EC_50_ values (concentration of which the half of the maximum adverse effect was detected) at a defined time point was calculated by the Graph Pad Prism Software from viability values using the sigmoidal, 4PL, x is log (concentration) mathematical model.

## Results

### Dose dependent effect on epithelial cells exposed to bacterial toxins

Three bacterial toxins Streptolysin O, Hygromycin B and Nigericin, which all have different attack points in the cell, were separately applied to a confluent cell layer of NuLi-1 cells and viability changes were determined after 24 and 48 h using the Prestoblue™ assay. In parallel, impedance was measured over a period of 48 h.

**Fig. 1:**
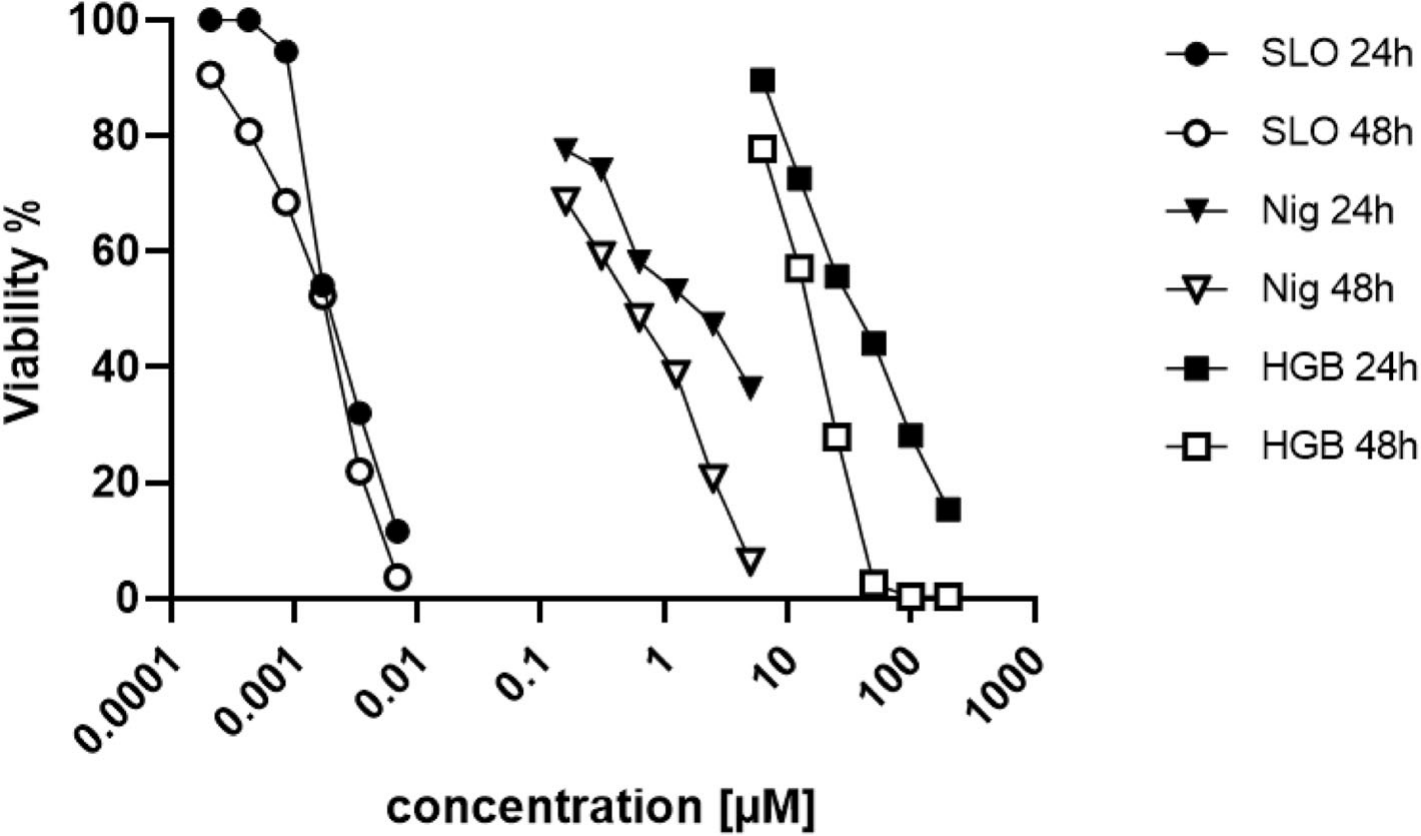
Viability of NuLi-1 cells exposed to bacterial toxins. Streptolysin O (circles), Nigericin (rectangles) and Hygromycin (squares) were applied in different concentrations to NuLi-1 cells and the viability was measured at 24 (filled symbols) and 48 h (empty symbols) using the PrestoBlue™ assay. Results are shown as a percentage of viable cells

All three toxins induced a dose-dependent cytotoxic effect to NuLi-1 cells, but at different concentrations (Fig. 1) as confirmed using the viability assay. Streptolysin O worked in a low nanomolar range (0.21–6.9 nM), Nigericin in a low micromolar range (0.16–5 μM) and Hygromycin B was toxic at higher μ-molar concentrations (8–252 μM). Half maximal effective concentration (EC50) of bacterial toxins were calculated for 24 or 48 h. The exposure data were used to plot a dose-response curve and calculate the EC50 values for each toxin (Table 2). The EC50 values for Streptolysin O of 0.002 μM for 24 h and 0.002 μM for 48 h are equal. In contrast, the EC50 values for Nigericin increased from 0.9 μM (24 h) to 1.1 μM after 48 h. The EC50 value of hygromycin B dropped from 40.3 μM (24 h) to 24.5 μM after 48 h. In summary, the EC50 calculation for the toxins showed that Hygromycin B is the toxin with the lowest toxicity, followed by Nigericin and Streptolysin O with the strongest toxicity.

**Table 2:** Calculated EC50 values (μM) of bacterial toxins after 24 and 48 h of exposure using viability data

	EC50 (μM)	
Toxin	24h	48h
Streptolysin O	0.002	0.002
Nigericin	0.9	1.1
Hygromycin B	40.3	24.5

**Fig. 2:**
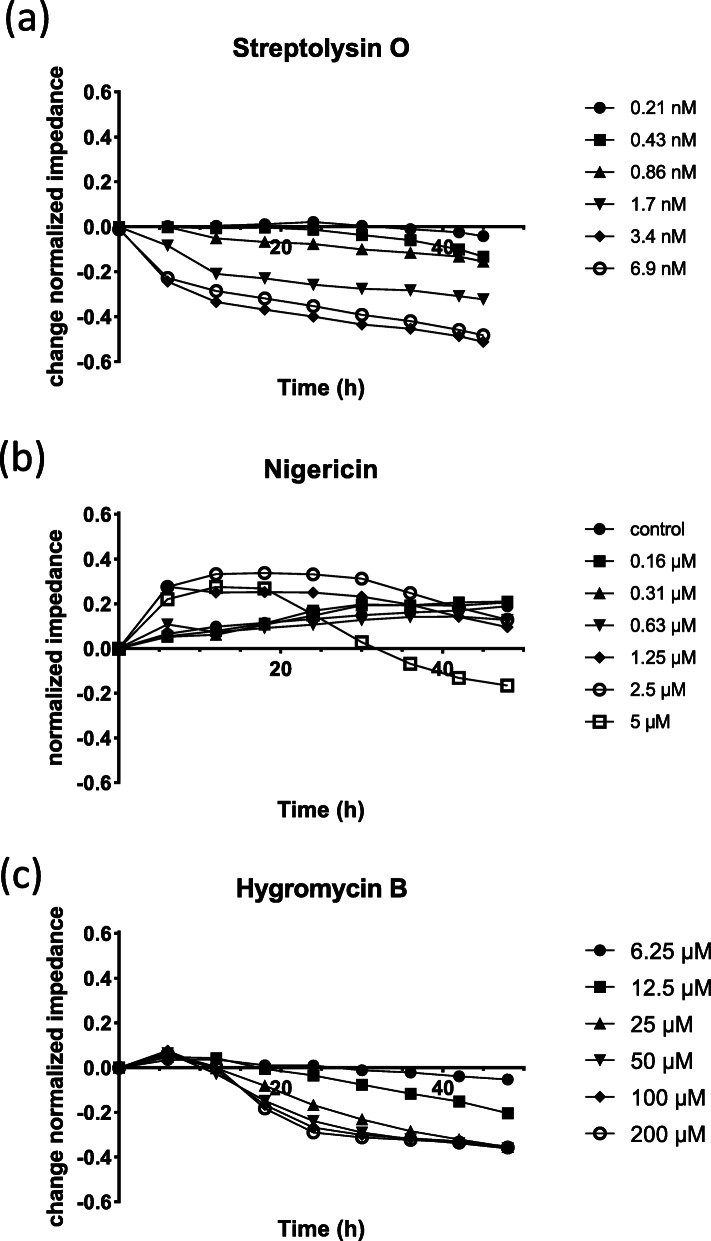
ECIS real-time monitoring of NuLi-1 cells in the presence of bacterial toxins. Streptolysin O (**a**), Nigericin (**b**) and Hygromycin (**c**) were applied at different concentrations to NuLi-1 cells and their effects on the impedance were measured over 48 h. Averages of normalized impedance differences between toxin exposed data and control data are shown from triplicates. Cell culture medium treated cells were used as controls. Impedance was normalized to cell values at the time of toxin addition

The impedance measurements showed that a decrease of the impedance values, i.e., loss in cell-cell contacts, i.e., loss in cell-adhesion-integrity was induced by all three toxins, but at different time-points, concentrations and in a different way (Fig. 2 a-c). Streptolysin O led to an impedance reduction in lung epithelial cells quite early after addition of the compound and impedance values dropped continuously until the end of measurement even at the lowest concentration. The time course showed that all concentrations induced to a decrease in impedance. The higher the concentration, the earlier the effect of streptolysin O began and the stronger the effect was. The impedance changes caused by Nigericin were induced immediately after addition of a concentration of 1.25 μM and higher. However, Nigericin led first to an increase in impedance, which then slowly decreased depending on the used concentration over time. The course of time of Hygromycin B, on the other hand, showed that all concentrations led to a decrease in impedance similar to that of Streptolysin O. But in comparison the effects appear much later and in addition the effect of Hygromycin B is much weaker than that of Streptolysin O. When looking at the impedance profiles, it is noticeable that the course of Streptolysin O and Hygromycin B is monophasic. The impedance profile of Nigericin showed a biphasic reaction characteristic with a higher impedance after 12 h, an equal impedance after 24 h and a lower impedance after 48 h.

### Dose dependent effect on epithelial cells exposed to a range of bacterial supernatants

After showing the dependence of pure substances bacterial culture supernatants were analyzed using the established assays. Cells were exposed to four dilutions of the supernatants from the stationary phase culture. The optimal dilutions for the representation of a dose-response relationship were determined in preliminary tests (data not shown) for each bacterium based on the early stationary phase culture.

**Fig. 3:**
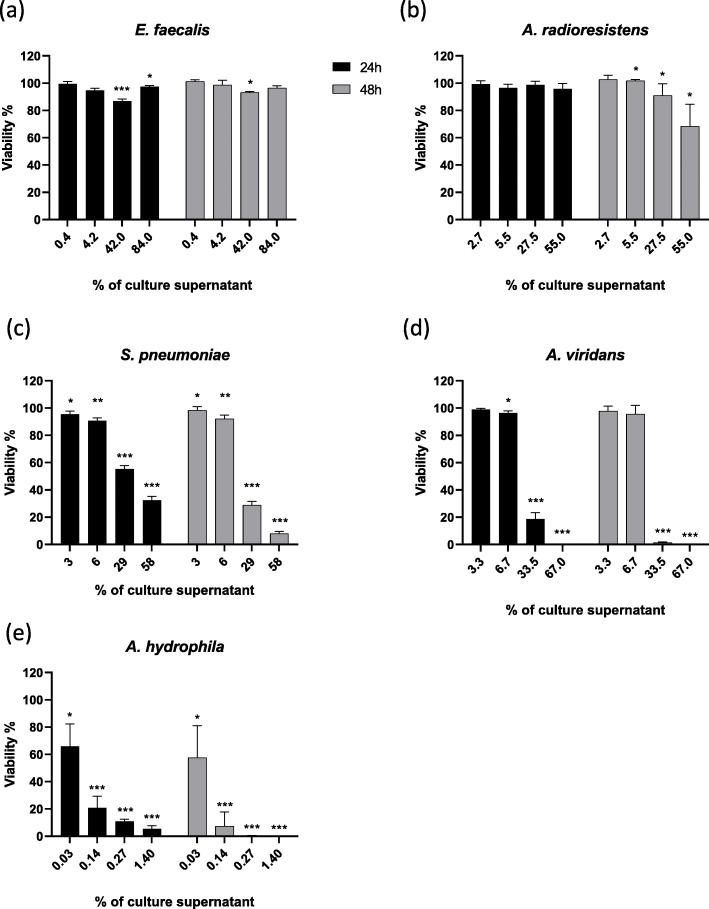
Viability of the NuLi-1 cells in the presence of bacterial culture supernatants. The collected bacterial supernatants of *E.faecalis* (**a**), *A.radioresistens* (**b**), *S.pneumoniae* (**c**), *A.viridans* (**d**) and *A.hydrophila* (**e**) were applied at four different concentrations (percentage of the culture supernatant in 100 μl sample) and their effects were measured at 24 (black bars) and 48 h (grey bars). Cells treated with bacterial growth medium THY were used as controls reflecting 100% cell viability. The results are shown as a percentage of viable cells. The asterisk indicates statistically significant differences compared to the control (* = *p* < 0.05, ** = *p* < 0.01, *** = *p* < 0.001)

The Prestoblue™ assay was used to determine cells viability using four dilutions (expressed as percentage of the culture supernatant in the sample) of bacterial supernatants at 24 h and 48 h respectively (Fig. 3 a-e). The results showed no decline in viability in the presence of the culture supernatant of *E. faecalis*. For *A. radioresistens*, there was a significant but low loss of viability after 48 h for the highest three concentrations of the culture supernatants. The supernatants of the other three bacteria *S. pneumoniae*, *A. viridans* and *A. hydrophila* showed a significant loss of viability already after 24 h. The EC50 values were calculated for 24 and 48 h after adding culture supernatants (Table 3). The EC50 values showed the lowest toxicity for *S. pneumoniae* (EC50_24h_ 34%; EC50_48h_ 19%), a strong toxicity for *A. viridans* (EC50_24h_ 21%; EC50_48h_ 11%) and the strongest toxicity for *A. hydrophila* (EC50_24h_ 0.1%; EC50_48h_ 0.1%). The EC50 values for *E. faecalis* and *A. radioresistens* could not be calculated.

**Table 3:** Calculate EC50 values (% of culture supernatants) of bacteria after 24 and 48 hours of exposure using viability data.

	EC50 (% culture supernatant)	
Bacteria	24h	48h
*E. faecalis*	-	-
*A. radioresistents*	-	-
*S. pneumoniae*	34	19
*A. viridans*	21	11
*A. hydrophila*	0.1	0.1

**Fig. 4:**
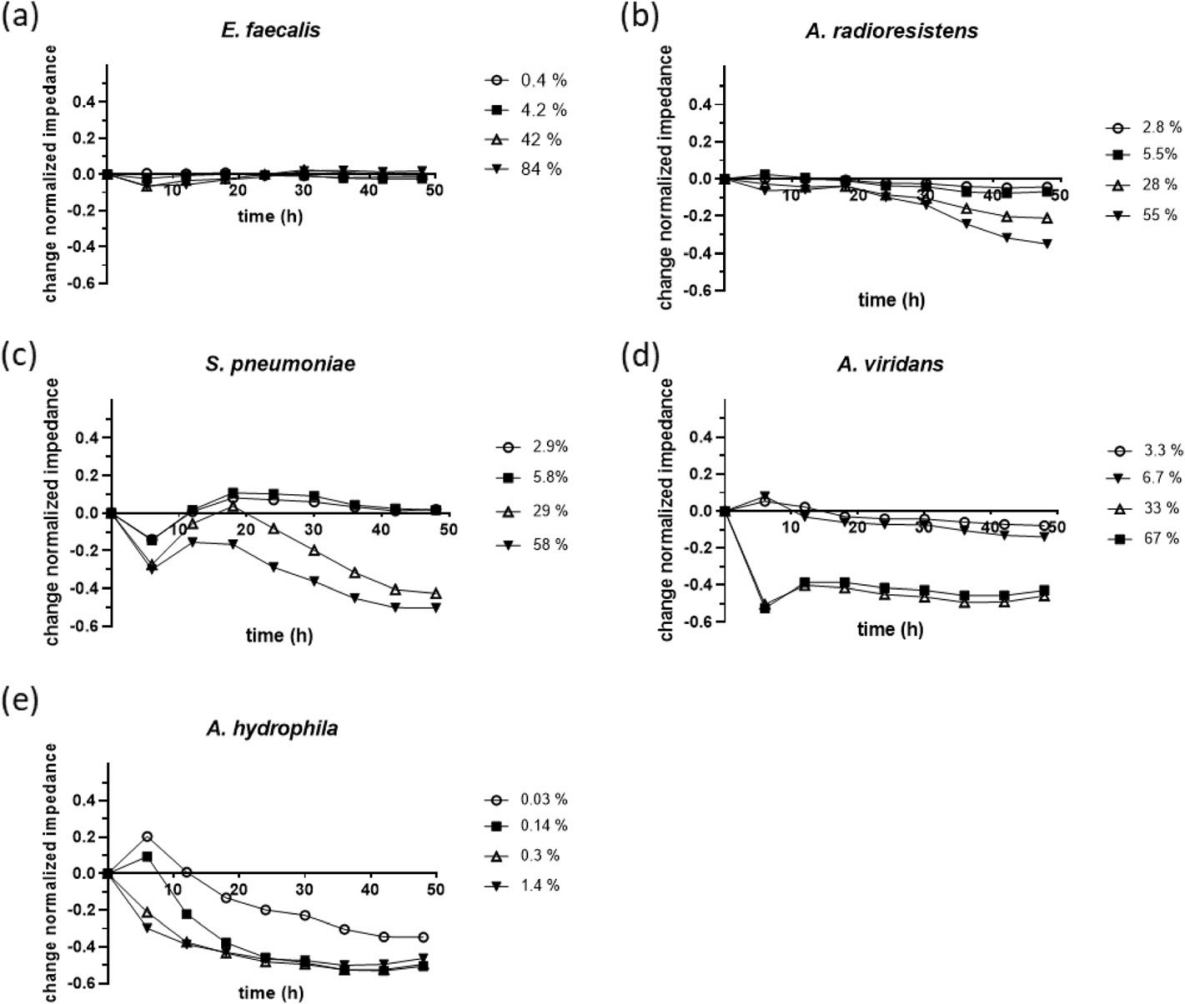
ECIS real-time monitoring of NuLi-1 cells in the presence of bacterial culture supernatants. Collected supernatants of the bacteria *E.faecalis* (**a**), *A.radioresistens* (**b**), *S.pneumoniae* (**c**), *A.viridans* (**d**) and *A.hydrophila* (**e**) were applied at four different concentrations (percentage of the culture supernatant in 100 μl sample) to NuLi-1 cells and their effects were measured over 48 h. Averages of normalized impedance differences between supernatant exposed data and control data are shown from 3 measured biological replicates. Cells exposed to bacterial growth medium THY were used as controls. Impedance was normalized to values at the time of supernatant addition

Additionally, the impedance measurement was used to monitor the cell changes of the NuLi-1 cells while exposed to the same dilutions of bacterial culture supernatants as in the viability assay over 48 h (Fig. 4 a-e). The results showed that *E. faecalis* (Fig. 4a) did not cause any change of NuLi-1 cells within 48 h in the feasible dilution range, since there was no change in impedance at any dilution tested compared to the control. For *A. radioresistens* the impedance in all four dilutions decreased slightly in the first 20 h. Afterwards the 28%- and 55% sample increasingly lost impedance whereas the lower concentrated supernatants constantly decreased over time. *S. pneumoniae* (Fig. 4c), on the other hand, showed first a slight increase followed by a continuous decrease in cell resistance directly from the beginning at the two highest concentrations. In contrast to *S. pneumoniae*, *A. viridans* showed a sudden drop in cell resistance from the beginning of the exposure, which remains the same for the rest of the measurement time. *A. hydrophila* showed a continuous decrease in cell resistance from the beginning of the exposure at the two highest concentrations, whereas the lower concentrations led first to an increase of the cell resistance followed by a continuous decrease over the measurement time. Thus, the data indicate that cytotoxicity potential was dependent on the quantity of toxic secondary metabolites of the bacterial culture supernatant, which could be distinguished using the ECIS system.

## Discussion and conclusion

Our results of the chosen test strategy indicate that the chosen toxins and culture supernatants of bacteria can influence NuLi-1 cell-layer formation and viability in a dose dependent manner, as indicated by the changes in impedance and viability values of the exposed cells compared to not exposed control cells.

The toxin impedance measurements showed a dose-dependent profile typical for each tested toxin and could therefore an appropriate method to distinguish the mechanisms of toxins.

Streptolysin O from *Streptococcus pyogenes* is a secreted protein of the group of thiol-activated cytolysins (TACY) [27]. The initial interaction of the TACY family is to bind cholesterol-containing membranes and then oligomerize to form transmembrane pores leading to subsequent a cell collapse [28]. This effect could be described very well by our impedance and viability measurements. The Streptolysin O impedance profile showed a rapid drop in impedance completed with a low viability of the cells measured after 24 and 48 h exposure. Hygromycin B is an antibiotic synthesized by *Streptomyces hygroscopicus*. It inhibits the protein biosynthesis leading to cell death [29, 30]. The Hygromycin B profile showed a delayed drop in cellular impedance due to inhibited protein biosynthesis. The cytotoxic effect of the inhibited protein synthesis takes some time until effects are measurable as cells can rely on their present available proteins in the cells. The third toxin Nigericin is an ionophore, also synthesized by *Streptomyces hygroscopicus*. Nigericin possess a high affinity to monovalent cations such as Na + and K+ and is known to disrupt the ionic balance, thereby affecting the transmembrane potential across the plasma membrane by actively transporting cations [31]. This in turn leads to an increased osmotic swelling and subsequent cell death due to bursting [32]. Furthermore, Nigericin might inhibit mitochondrial ATP synthesis by changing the intracellular K+ and H+ concentrations, which may change the electrochemical gradient across the inner membrane of mitochondria [33]. The Nigericin impedance profile showed first an increased impedance typical to an osmotic swelling of the cells followed by a decrease in impedance due to the bursting of the cells, what could be also observed microscopically (data not shown).

The effect analysis was also done for culture supernatants of selected bacteria. Based on the literature, we expected cytotoxic effects for *E. faecalis* [34], *S. pneumoniae* [35, 36], *A. hydrophila* [37, 38]; and *A. radioresistens* [39]. For *E. faecalis* we could not specify any effects using both methods. This could mean that either, the toxin cannot attack lung epithelial cells, as NuLi-1 or the toxin is not present in sufficient amounts or in an insoluble form in the culture supernatant under our chosen laboratory conditions. Earlier studies by Todd showed that hemolytic *E. faecalis* strains isolated on blood agar exhibited no longer hemolytic activity after cultivation in standard liquid medium. Now, one assumes that the formed cytolysine is present in insoluble form in the medium and therefore no effect is detectable [25]. Since we were not able to detect hemolysis on blood agar of tested culture supernatants of *E. faecalis* (data not shown), we assume that the hypothesis of a deficient cytolysine production was the reason for the missing effects on the NuLi-1 cells. The production of toxins depends on the selected laboratory conditions like media utilized for cultivation. In order to establish this assay as a standard method, several media should be included in the future. In addition, supporting methods for identifying toxins such as protein identification by mass spectrometry could complement the characterization of tested bacteria. For *A. radioresistens*, it has been shown, that outer membrane vesicles (OMVs) cause a dose-dependent cytotoxicity in HEK-293 cells possibly by inducing apoptosis [24]. Additionally, an OmpA-like protein was identified in OMV fraction; OmpA is already known as a cytotoxic protein secreted from *Acinetobacter baumanii* [22]. Our analysis of *A. radioresistens* with both methods showed a slight cytotoxic effect on the NuLi-1 cells. This occurs recognizably by the ECIS measurement after approximately 36 h of exposure and also resulted in a loss of viability of approx. 30% in the 48-h measurement of the viability measurement. Thus, it is quite possible that *A. radioresistens* produced cytotoxic outer membrane proteins like the OmpA-like protein or the cytotoxic Lipid A as already published. As expected, *S. pneumoniae* and *A. hydrophila* showed also a strong cytotoxic effect using the viability assay and physiological effects using the impedance measurement on the NuLi-1 cells. *S. pneumoniae* has many known virulence factors like the hyaluronidase and pneumolysin. Pneumolysin belongs like Streptolysin O to the group of thiol-activated cytolysins and can directly damage the host cell leading to lysis [36]. *Aeromonas* infections present a variety of clinical manifestations triggered by some virulence factors. Extracellular proteins leading to host cell damage are cytotoxic enterotoxins, hemolysins and proteases [40]. Furthermore, we showed for the first time a strong cytotoxic effect of *A. viridans* on lung epithelial cells. From the literature, *A. viridans* is known as an inducer of a robust inflammatory reaction and a strong cytotoxic effect in contact with cultured bovine mammary epithelial cells [41]. *Aerococcus* is often identified as (bio) indicator organism in bioaerosol studies in poultry and pig plants [42, 43] and is therefore an interesting pathogen in the field of occupational safety and health.

Our data show that both methods in combination used in this study are suitable for describing bacterial toxicities on human lung epithelial cells. The viability test as an endpoint assay describes the cytotoxic effect and allows the description of a toxic bacterium based on the EC50 value. The impedance measurement, in turn, reflects real-time effects in the presence of toxic bacteria on lung epithelial cells. Difference in the mode of action of the used toxins and bacteria on the Nuli-1 cells have to be considered. Even of many cellular activities, such as cell adhesion, spreading, proliferation, physiological activity and different ways to cell death influence the impedance of the cells. Dynamic monitoring of the cell response to a microbiological trigger, measured by ECIS has one clear advantage: compared to conventional endpoint assays that measure one endpoint, it allows a monitoring of the overall cell behavior over time. In addition, it is easy to check whether toxic effects are reversible and/or limitations are maintained in the cell after exposure. Furthermore, it is a label-free method and proves to be a rapid diagnostic tool for analysis of cellular behavior in cell-based in vitro assays. Classical endpoint assays just measure e.g., viability at an endpoint but do not allow considerations on the toxic influence on cells before.

To conclude, even without an extensive analysis cell-based sensor like ECIS would be a powerful tool in combination with a viability endpoint assay, when quick statements about toxicity potentials are needed for the risk assessment of workplaces. In addition, impedance profiles increase the probability for more information about the onset, mode of action and the time course of the toxicity and the ECIS system can be used as future rapid diagnostic tool to indicate health adverse effects to the inhalation of bacteria at workplaces.

## Data Availability

Not applicable.

## Abbreviations

ECIS: Electrical cell-substrate impedance sensing
PB: Prestoblue™
SLO: Streptolysin O
HGB: Hygromycin B
N: Nigericin
THY: Todd Hewitt Broth + 0.5% yeast extract medium
AECBM: Serum-free airway epithelial cell-base medium
EC50: Half maximal effective concentration
cfu: Colony-forming units
hTERT: Human telomerase reverse transcriptase
